# Erratum to: *Pilot and Feasibility Studies*, Vol. 4

**DOI:** 10.1186/s40814-017-0183-2

**Published:** 2017-10-24

**Authors:** Pilot and Feasibility Studies

**Affiliations:** London, UK

## Erratum

An error occurred during the publication of a number of articles in *Pilot and Feasibility Studies*. These articles were erroneously published in volume number 4, which is listed with a publication date of 2018. However, these articles were published in final form in the year 2017.

Provided below are the actual publication dates and the correct citation for each affected article. Please note that the citation refers to volume number 4 with the year as 2018.

Article: 10.1186/s40814-017-0168-1

Publication date: 27 July 2017.

Correct citation: Gunn et al. *Pilot and Feasibility Studies* (2018) 4:26. 10.1186/s40814-017-0168-1

Article: 10.1186/s40814-017-0167-2

Publication date: 20 July 2017.

Correct citation: Smith et al. *Pilot and Feasibility Studies* (2018) 4:24. 10.1186/s40814-017-0167-2

Article: 10.1186/s40814-017-0161-8

Publication date: 20 July 2017.

Correct citation: Cruickshank et al. *Pilot and Feasibility Studies* (2018) 4:22. 10.1186/s40814-017-0161-8

Article: 10.1186/s40814-017-0166-3

Publication date: 20 July 2017.

Correct citation: Cadogan et al. *Pilot and Feasibility Studies* (2018) 4:23. 10.1186/s40814-017-0166-3

Article: 10.1186/s40814-017-0169-0

Publication date: 20 July 2017.

Correct citation: Mitchell et al. *Pilot and Feasibility Studies* (2018) 4:25. 10.1186/s40814-017-0169-0

Article: 10.1186/s40814-017-0170-7

Publication date: 18 July 2017.

Correct citation: Price et al. *Pilot and Feasibility Studies* (2018) 4:21. 10.1186/s40814-017-0170-7

Article: 10.1186/s40814-017-0171-6

Publication date: 17 July 2017.

Correct citation: Davies et al. *Pilot and Feasibility Studies* (2018) 4:20. 10.1186/s40814-017-0171-6

Article: 10.1186/s40814-017-0158-3

Publication date: 17 July 2017.

Correct citation: Best et al. *Pilot and Feasibility Studies* (2018) 4:18. 10.1186/s40814-017-0158-3

Article: 10.1186/s40814-017-0165-4

Publication date: 14 July 2017.

Correct citation: Schick et al. *Pilot and Feasibility Studies* (2018) 4:19. 10.1186/s40814-017-0165-4

Article: 10.1186/s40814-017-0160-9

Publication date: 10 July 2017.

Correct citation: Arikawa et al. *Pilot and Feasibility Studies* (2018) 4:17. 10.1186/s40814-017-0160-9

Article: 10.1186/s40814-017-0157-4

Publication date: 7 July 2017.

Correct citation: Kemp et al. *Pilot and Feasibility Studies* (2018) 4:16. 10.1186/s40814-017-0157-4

Article: 10.1186/s40814-017-0163-6

Publication date: 7 July 2017.

Correct citation: Lennox et al. *Pilot and Feasibility Studies* (2018) 4:15. 10.1186/s40814-017-0163-6

Article: 10.1186/s40814-017-0162-7

Publication date: 6 July 2017.

Correct citation: Huberty et al. *Pilot and Feasibility Studies* (2018) 4:12. 10.1186/s40814-017-0162-7

Article: 10.1186/s40814-017-0152-9

Publication date: 6 July 2017.

Correct citation: Armor et al. *Pilot and Feasibility Studies* (2018) 4:10. 10.1186/s40814-017-0152-9

Article: 10.1186/s40814-017-0164-5

Publication date: 6 July 2017.

Correct citation: Cooper et al. *Pilot and Feasibility Studies* (2018) 4:13. 10.1186/s40814-017-0164-5

Article: 10.1186/s40814-017-0154-7

Publication date: 6 July 2017.

Correct citation: Shepherd et al. *Pilot and Feasibility Studies* (2018) 4:11. 10.1186/s40814-017-0154-7

Article: 10.1186/s40814-017-0156-5

Publication date: 6 July 2017.

Correct citation: Jadczak et al. *Pilot and Feasibility Studies* (2018) 4:8. 10.1186/s40814-017-0156-5

Article: 10.1186/s40814-017-0153-8

Publication date: 6 July 2017.

Correct citation: Heckman et al. *Pilot and Feasibility Studies* (2018) 4:9. 10.1186/s40814-017-0153-8

Article: 10.1186/s40814-017-0150-y

Publication date: 6 July 2017.

Correct citation: Twohig et al. *Pilot and Feasibility Studies* (2018) 4:7. 10.1186/s40814-017-0150-y

Article: 10.1186/s40814-017-0159-2

Publication date: 6 July 2017.

Correct citation: Thabane and Lancaster *Pilot and Feasibility Studies* (2018) 4:14. 10.1186/s40814-017-0159-2Article: 10.1186/s40814-017-0147-6

Publication date: 21 June 2017.

Correct citation: Joseph et al. *Pilot and Feasibility Studies* (2018) 4:5. 10.1186/s40814-017-0147-6

Article: 10.1186/s40814-017-0148-5

Publication date: 21 June 2017.

Correct citation: Leach et al. *Pilot and Feasibility Studies* (2018) 4:6. 10.1186/s40814-017-0148-5

Article: 10.1186/s40814-017-0155-6

Publication date: 20 June 2017.

Correct citation: Stone and Ndagijimana *Pilot and Feasibility Studies* (2018) 4:4. 10.1186/s40814-017-0155-6

Article: 10.1186/s40814-017-0151-x

Publication date: 17 June 2017.

Correct citation: Jones *Pilot and Feasibility Studies* (2018) 4:3.

10.1186/s40814-017-0151-x

Article: 10.1186/s40814-017-0149-4

Publication date: 15 June 2017.

Correct citation: Nguyen et al. *Pilot and Feasibility Studies* (2018) 4:2. 10.1186/s40814-017-0149-4

Article: 10.1186/s40814-017-0145-8

Publication date: 9 June 2017.

Correct citation: Learmonth and Motl *Pilot and Feasibility Studies* (2018) 4:1. 10.1186/s40814-017-0145-8

Since this error was detected, the journal has resumed publication in the 2017 volume, volume number 3. In 2018, the journal will resume publication in volume number 4. The publisher apologizes for any confusion caused by this error.

